# Genetically Validated Immune Susceptibility Markers for Crohn's Disease: A Multi‐Omics Mendelian Randomization Analysis

**DOI:** 10.1002/jgh3.70332

**Published:** 2026-01-01

**Authors:** Zeyang Li

**Affiliations:** ^1^ School of Physical Education Qilu Normal University Jinan Shandong China

**Keywords:** Crohn's disease, HLA‐A, immune susceptibility marker, Mendelian randomization

## Abstract

**Background:**

Crohn's disease (CD) is a chronic inflammatory bowel disorder with a multifactorial genetic, immune, and environmental basis, yet robustly validated therapeutic targets remain limited. We applied a multi‐omics Mendelian randomization (MR) framework and colocalization to prioritize genetically supported immune susceptibility loci for CD.

**Methods:**

A two‐sample MR framework was used, integrating protein quantitative trait loci (pQTL) and expression quantitative trait loci (eQTL) datasets with genome‐wide association study (GWAS) data on CD from the Finnish biobank. After identifying 994 pQTL genes, 610 genes overlapping with known druggable targets were selected. Causal associations with CD were evaluated using inverse variance weighted (IVW) MR. Significant hits were further validated through colocalization analysis and summary‐data‐based Mendelian randomization (SMR).

**Results:**

IVW analysis identified 56 pQTL genes significantly associated with CD risk, with six genes (including *HLA‐A*, *MST1*, and *CDH5*) passing false discovery rate (FDR) correction. Colocalization analysis indicated that *HLA‐A* and *MST1* shared causal variants with CD. Subsequent eQTL‐based MR and SMR analysis confirmed the causal association of *HLA‐A* expression with increased CD risk (SMR OR = 1.434, *p* = 4.10 × 10^−5^), with no evidence of heterogeneity.

**Conclusion:**

These findings suggest that HLA‐A represents a genetically validated immune susceptibility marker in CD. By integrating pQTL, eQTL, colocalization, and SMR analyses, it highlights the utility of multi‐omics MR in uncovering novel genetic contributors to complex diseases. Further experimental and clinical validation is warranted to explore the translational potential of targeting *HLA‐A* in CD treatment.

## 1 Introduction

Crohn's disease (CD) is a chronic inflammatory bowel disease (IBD) characterized by nonspecific inflammation of the gastrointestinal tract, affecting any part from the mouth to the anus [1], with the small intestine and colon being the most commonly involved areas [2]. The disease typically presents with abdominal pain, diarrhea (often with blood), weight loss, decreased appetite, and systemic symptoms such as fatigue and fever [3, 4]. Epidemiological data indicate significant variations in incidence rates of the disease across different regions [5]. The incidence is higher in North America and Europe, reaching 10–20 cases per 100,000 per year, whereas it is relatively lower in Asia [6]. Studies have shown that globally, the number of individuals affected by CD exceeds 3 million, particularly prevalent among young adults aged 15–30 [2]. The pathophysiological mechanisms of CD are not yet fully elucidated, but it is widely believed to result from the interplay of multiple factors.

Abnormal immune responses are considered a significant factor in the onset of the disease. Immune cells in the body react excessively to the normal gut microbiota, resulting in intestinal inflammation [7, 8]. Second, genetic factors are also believed to play an important role in the development of CD. The familial clustering of CD suggests a potential genetic susceptibility. Research indicates that approximately 20% of patients with CD have a family history of the condition [9]. The genome‐wide association studies (GWASs) have shown that gene loci associated with oxidative stress may be linked to CD [10, 11]. Furthermore, DNA methylation (DNAm) regulates intestinal immunity and inflammatory responses by modulating the gene expression of NRF2, HIF1A, and related proteins [12, 13]. Environmental factors such as dietary habits, smoking, and the use of certain medications may also exacerbate the condition [14]. Therefore, identifying new drug targets to improve treatment strategies for CD has become a crucial focus of current research.

Mendelian randomization (MR) uses genetic variations associated with relevant exposures as instrumental variables (IVs) to assess their causal effects on outcomes [15, 16]. MR Since genetic variations are considered to be randomly allocated, they are not influenced by the widespread confounding factors and reverse causation often encountered in observational analyses [17]. MR analysis can identify immune susceptibility markers associated with the pathogenesis of CD by examining the relationship between genetic variations and disease risk. Moreover, MR analysis integrates genomic data from various sources, enhancing the accuracy and reliability of target identification.

Drug targets refer to specific molecules or cellular structures in the body that drugs interact with, typically including proteins, nucleic acids, or other biomolecules [18]. These targets play a crucial role in the physiological and pathological processes of cells, and drugs exert their therapeutic effects by interacting with these targets to regulate biological processes. The mechanisms of action of drug targets can be achieved through various means, including enzyme inhibition, receptor modulation, and transcription factor regulation. For instance, nonsteroidal anti‐inflammatory drugs (NSAIDs) reduce inflammation and pain by inhibiting cyclooxygenase (COX) [19]. The pathogenesis of CD is complex, and identifying and targeting appropriate drug targets is essential for developing effective treatments. With the advancement of genomics, MR analysis based on druggable genes provides new insights for immune susceptibility markers in CD.

## 2 Methods

**FIGURE 1:**
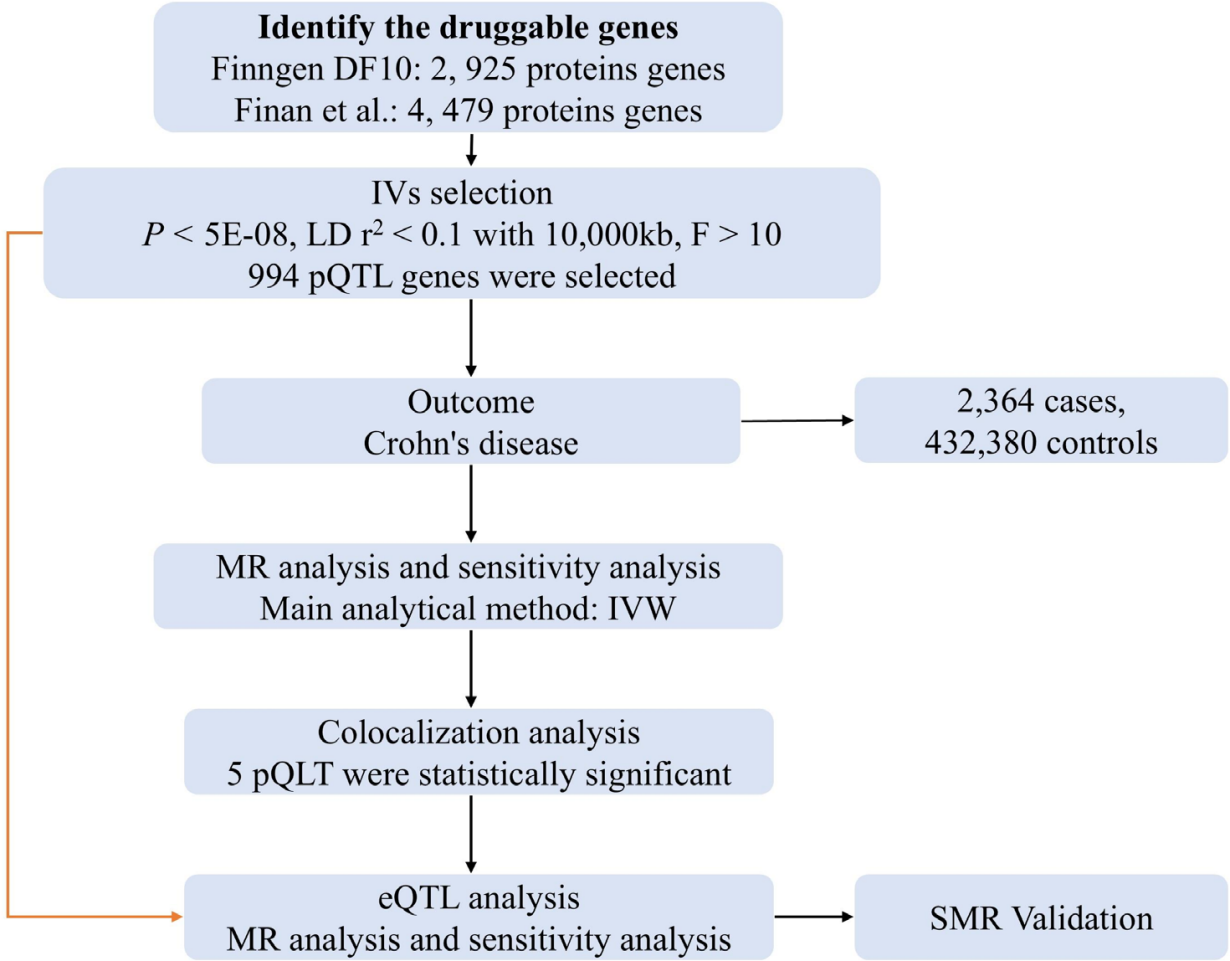
The flowchart of study design. CD, Crohn's disease; eQTL, expression quantitative trait locus; MR, Mendelian randomization; pQTL, protein quantitative trait locus.

In this study, we used a two‐sample MR analysis as the primary research method to identify immune susceptibility markers associated with CD. The study design initially used the latest protein quantitative trait locus (pQTL) data from the Finnish database as exposure, while the large‐scale GWAS data on CD served as the outcome to assess the causal relationship between pQTL and CD. Colocalization analyses were performed for drugable genes with significant causal effects. Subsequently, MR analysis of expression quantitative trait loci (eQTL) with CD was performed to initially validate the selected druggable genes. Although blood is an informative tissue for immune‐mediated traits, large‐scale multi‐tissue efforts have demonstrated that many *cis*‐eQTLs exhibit marked tissue specificity, including in gastrointestinal tissues [20]. Finally, we used summary‐data‐based Mendelian randomization (SMR) to further validate a series of selected genes. Figure 1 illustrates the fundamental workflow of this study.

### 2.1 Data Sources

#### 2.1.1 pQTL Data

The new pQTL data are derived from the Finnish biobank study [21]. The DF10 version of the data comprises samples from 412 181 Finns, released on December 18, 2023. Among these, the pQTL data includes 619 samples, covering data on 2925 proteins. In the original study, the FinnGen study protocol was approved by the Coordinating Ethics Committee of the Hospital District of Helsinki and Uusimaa (HUS) (approval number HUS/990/2017).

#### 2.1.2 eQTL Data

According to research by Finan et al., a total of 4479 druggable genes have been identified, including 1427 genes encoding approved drug targets or those in clinical trials, 682 genes encoding proteins that can bind to known drug molecules or are similar to approved drug targets, and 2370 genes that belong to key druggable gene families or encode proteins with more distant similarities to approved drug targets [22]. The eQTL data are publicly available, and the genomic data for eQTLs are derived from multiple open‐source databases. Therefore, no additional ethical approval is required. In the present analysis, *cis*‐eQTL instruments for these druggable genes were derived from blood‐based eQTL summary statistics. Thus, the eQTL data used here primarily reflect gene expression regulation in peripheral blood rather than in intestinal tissues such as colon or ileum.

#### 2.1.3 CD Data

The CD data is derived from the DF11 version of the Finnish biobank study, released on June 24, 2024 [21]. CD is defined as a gastrointestinal disorder characterized by chronic inflammation involving all layers of the intestinal wall, noncaseating granulomas affecting the intestinal wall and regional lymph nodes, and transmural fibrosis. The dataset includes 434 744 participants (2364 patients and 432 380 controls). In the original study, the FinnGen study protocol was approved by the Coordinating Ethics Committee of the Hospital District of Helsinki and Uusimaa (HUS) (approval number HUS/990/2017).

### 2.2 IV Selection

In MR analysis, the SNPs used as IVs must meet the following three assumptions: Assumption 1: IVs must exhibit a strong and robust correlation with the exposure factor (relevance assumption) [23]. Assumption 2: IVs must not be directly related to the outcome variable, influencing the outcome only through the exposure factor (exclusivity assumption) [24]; Assumption 3: IVs must not be associated with other confounding factors that influence the “exposure‐outcome” relationship (independence assumption) [25]. First, we applied a stringent threshold to filter significant SNPs from the pQTL dataset (*p* < 5 × 10^−8^). Subsequently, we removed palindromic SNPs and those with uncertain directions using a linkage disequilibrium (LD) clustering method, ultimately obtaining independent SNPs as IVs (with a genetic distance set at 10000 kb and an LD parameter *r* ^2^ = 0.001). Finally, remove IVs with *F* < 10 [26].

### 2.3 MR Analysis

IVW is the primary method used to evaluate the effects of MR results [27]. IVW first calculates the Wald ratio for each IV between exposure and outcome, then performs a meta‐analysis of all IVs' Wald ratios to estimate the gene‐predicted causal relationship [28]. The WME provides robust causal effect estimates even when up to 50% of the IVs are invalid, though it may increase the error in the results [29]. MR‐Egger is used to detect and correct for bias caused by horizontal pleiotropy, although its statistical power is relatively low [30, 31]. The simple mode method disregards the direct effects of SNPs on the outcome and focuses solely on the impact of SNPs on the exposure and the effect of exposure on the outcome. The weighted mode method assigns weights to the gene‐exposure and gene‐outcome relationships based on the effect sizes of SNPs on the outcome [32, 33].

### 2.4 Colocalization

We conducted colocalization analysis using the R package “coloc” (version 5.1.0.1) [34]. The default prior probabilities were set as follows: *p*_1_ = 1 × 10^−4^ (the prior probability that a SNP is associated with gene expression), *p*_2_ = 1 × 10^−4^ (the prior probability that the SNP is associated with the outcome), and *p*_12_ = 1 × 10^−5^ (the prior probability that the SNP is associated with both gene expression and the outcome). The colocalization analysis generated posterior probabilities for five hypotheses: PPH0, where the SNP is not associated with either gene expression or the outcome; PPH1, where the SNP is associated with gene expression but not with the outcome; PPH2, where the SNP is associated with the outcome but not with gene expression; PPH3, where the SNP is associated with both gene expression and the outcome, but with different causal variants; and PPH4, where the SNP is associated with both gene expression and the outcome, sharing the same causal variant. A posterior probability for PPH4 greater than 0.80 was considered strong evidence of colocalization. For each gene, the variant most strongly associated with the exposure (i.e., the variant with the lowest *p* value) was selected as the index variant, and all variants within ±500 kb of this index variant were included in the colocalization analysis.

### 2.5 SMR

SMR is a statistical approach that integrates GWAS and eQTL summary data to detect causal associations between gene expression levels and complex traits or diseases [12]. SMR analysis was conducted using specialized SMR software, following standard analytical procedures. The heterogeneity in dependent instruments (HEIDI) test was performed using multiple SNPs within the region to distinguish effects caused by shared genetic variants. By incorporating the SMR method, we were able to further validate the results of the MR and colocalization analyses, enhancing the reliability and robustness of our findings.

## 3 Result

After LD screening, a total of 994 pQTL genes were extracted for batch MR analysis. After taking the intersection of the pQTL genes with the druggable genes, 610 pQTL genes were obtained. The *F* statistics for the IVs were all > 20, indicating that there were no weak IVs (Table S1).

### 3.1 MR Analysis

**FIGURE 2:**
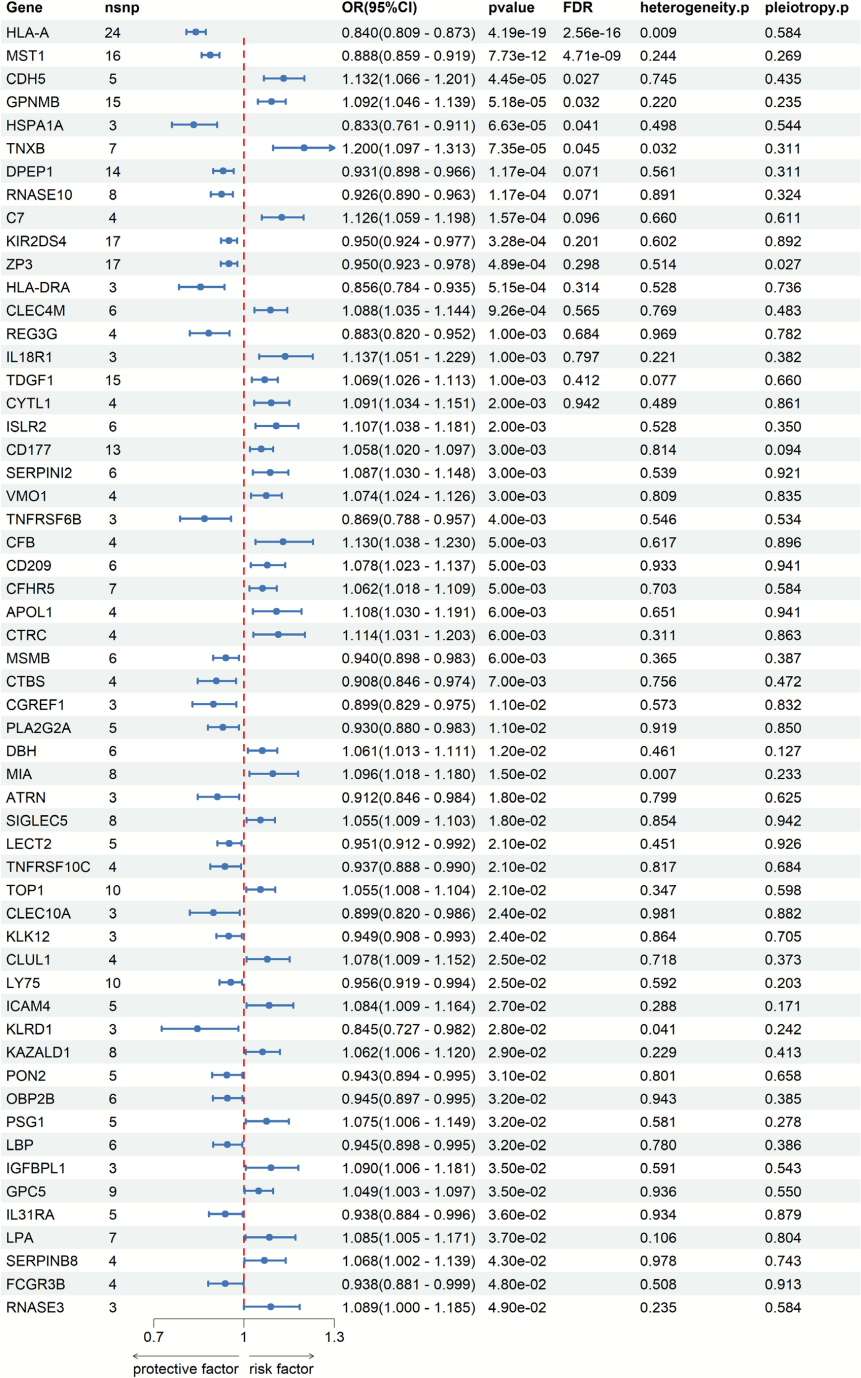
MR results of pQTL and CD. OR, odds ratio; SNP, single nucleotide polymorphism.

IVW results show that the expression of 56 pQTL genes was significantly causally associated with the risk of CD (*p* < 0.05) (Figure 2 and Table S2). Among these, the expression of six pQTL genes remained significant after false discovery rate (FDR) correction (*p*_FDR_ < 0.05), including *HLA‐A* (OR: 0.840, 95% CI: 0.809–0.873, *p*_FDR_ = 2.56 × 10^−16^), *MST1* (OR: 0.888, 95% CI: 0.859–0.919, *p*_FDR_ = 4.71 × 10^−09^), *CDH5* (OR: 1.132, 95% CI: 1.066–1.201, *p*_FDR_ = 0.027), *GPNMB* (OR: 1.091, 95% CI: 1.046–1.139, *p*_FDR_ = 0.032), *HSPA1A* (OR: 0.833, 95% CI: 0.761–0.911, *p*_FDR_ = 0.040), and *TNXB* (OR: 1.200, 95% CI: 1.096–1.313, *p*_FDR_ = 0.045). Sensitivity analysis indicated heterogeneity in the results for *MIA*, *TNXB*, and *KLRD1* (*p* < 0.05). The intercept test of the MR‐Egger regression method revealed horizontal pleiotropy for the genes *IL‐18R1*, *RNASE3*, *TNXB*, *DBH*, *CLUL1*, *CGREF1*, and *LY75* in relation to the outcomes of CD (*p* > 0.05) (Table S2).

### 3.2 Colocalization

For pQTL genes with significant MR results, we conducted colocalization analysis to calculate the probability that pQTL and CD outcomes share causal variants. The colocalization analysis results suggest that CD and two pQTL genes (*HLA‐A* and *MST1*) may share a causal variant (Table S3). The H4 values were > 0.80% (*HLA‐A*: 99.99% and *MST1*: 99.49%). Therefore, based on the MR and colocalization analyses, *HLA‐A* and *MST1* have been identified as genetically supported candidate loci related to CD pathogenesis, which may inform future drug target discovery.

### 3.3 MR Analysis of eQTL and CD

**FIGURE 3:**
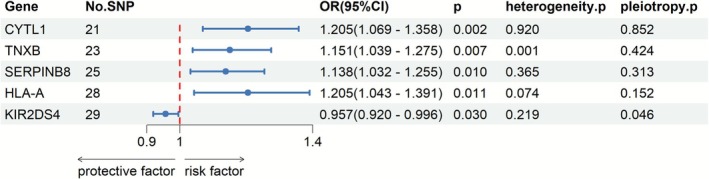
MR results of eQTL and CD.

The selection of druggable genes from the eQTL data included those that were significant in pQTL analysis and colocalization, with the IV selection criteria being the same as before (Table S4). The MR results for HLA‐A and MST1 in the eQTL data indicated that only *HLA‐A* significantly affected the risk of CD (OR: 1.205, 95% CI = 1.043–1.391, *p* = 0.011) (Figure 3 and Table S5). We determined that high levels of HLA‐A are significantly associated with increased risk of CD.

### 3.4 SMR Analysis

Finally, we used SMR software to further analyze the causal association between the identified *HLA‐A* gene and CD. The SMR results showed that *HLA‐A* significantly increased the risk of CD (OR: 1.434, 95% CI = 1.207–1.704, *p* = 4.10 × 10^−5^), and the HEIDI results indicated no heterogeneity (*p* = 0.251 > 0.05) (Table S6). In contrast, the MR associations for *MST1*, *CDH5*, *GPNMB*, *HSPA1A*, and *TNXB* were weaker did not all meet our prespecified colocalization or SMR thresholds; therefore, *HLA‐A* was prioritized for in‐depth interpretation, and the other proteins are considered hypothesis‐generating candidates. Notably, whereas higher genetically predicted plasma *HLA‐A* protein levels appeared to be associated with a reduced risk of CD, higher genetically predicted *HLA‐A* expression in blood and SMR estimates suggested increased risk, indicating a complex and potentially layer‐specific relationship between *HLA‐A* and CD susceptibility.

## 4 Discussion

This study identified *HLA‐A* as a genetically validated immune susceptibility locus for CD through large‐scale MR analysis, incorporating pQTL data, eQTL data, colocalization analysis, and SMR analysis, suggesting its important role in the pathogenesis of CD.

Beyond HLA‐A, previous genetic and translational studies have established several CD loci as key components of druggable immune pathways, particularly *IL‐23R*, *NOD2*, *TYK2*, and *JAK2*. Variants in *IL‐23R* and *NOD2* strongly influence IBD susceptibility, and recent work has highlighted how targeting the *IL‐23*/*Th17* axis can translate these findings into effective biologic therapies in CD and ulcerative colitis [35, 36]. In parallel, JAK–STAT signaling molecules such as *JAK2* and *TYK2* are enriched for IBD risk alleles, and selective *JAK* or *TYK2* inhibitors are now approved or in late‐phase clinical trials for luminal CD and UC [35, 37]. To place our multi‐omics MR findings within this therapeutic landscape, we therefore summarized these established CD drug targets alongside the six immune susceptibility markers identified in our analysis (*HLA‐A*, *MST1*, *CDH5*, *GPNMB*, *HSPA1A*, and *TNXB*) in Table 1.

**TABLE 1:** Comparison of CD therapeutic targets and candidate genes in MR analysis.

Gene	Key pathway/biological role	Therapeutic status in CD
*IL‐23R*	*IL‐23*/*Th17* signaling; strong CD/IBD risk locus *IL‐23*/*Th17*	*IL‐12*/*23* and *IL‐23* antibodies approved/late‐phase
*NOD2*	Bacterial sensing; innate immunity; landmark CD gene	No direct drug; informs microbial/innate‐immune strategies
*TYK2*	JAK kinase downstream of *IL‐12*/*23* and Type I IFNs	Selective *TYK2* inhibitors in Phase II–III IBD trials
*JAK2*	JAK–STAT signaling for multiple proinflammatory cytokines	JAK pathway inhibitors approved/in development for CD/UC
*HLA‐A*	MHC Class I antigen presentation; immune susceptibility locus	Genetically validated susceptibility marker; not directly drugged
*MST1*	Macrophage‐stimulating protein; innate‐immune regulation	No approved therapy; potential innate‐immune modulator
*CDH5*	VE‐cadherin; gut–vascular barrier integrity	Highlights vascular barrier as a candidate target
*GPNMB*	Myeloid cell glycoprotein; dampens mucosal inflammation	Preclinical protective factor in colitis models
*HSPA1A*	Inducible HSP70; epithelial stress response and repair	Heat‐shock pathways explored; no *HSPA1A*‐specific drug
*TNXB*	Extracellular matrix remodeling; intestinal fibrosis	ECM/fibrosis‐related locus; potential anti‐fibrotic target

*Note:* The table summarizes key pathways/biological roles and current therapeutic development status for selected established CD/IBD targets and for candidate genes highlighted by our study screening. Therapeutic status was summarized based on recent literature.

Abbreviations: CD, Crohn's disease; IBD, inflammatory bowel disease; IFN, interferon; MR, Mendelian randomization; pQTL, protein quantitative trait locus.

The *HLA‐A* gene is an important component of the human leukocyte antigen (*HLA*) complex, located on chromosome 6 [38]. *HLA* molecules are the main components of the major histocompatibility complex (MHC) and are involved in regulating the immune response of the body [39]. The *HLA‐A* gene encodes the *HLA‐A* protein, which primarily belongs to class MHC I molecules, responsible for presenting endogenous antigens to *CD8* ^+^ cytotoxic T lymphocytes, thereby activating cellular immune responses [40]. *HLA‐A* molecules also play a significant role in maintaining immune tolerance and self‐recognition [41]. Studies have shown that the polymorphisms of *HLA‐A* are associated with susceptibility to various autoimmune and infectious diseases [42].

*HLA‐A* gene is closely associated with disease susceptibility. For instance, studies have shown that specific *HLA‐A* alleles are linked to the occurrence of autoimmune diseases such as systemic lupus erythematosus (SLE) and rheumatoid arthritis [43, 44]. These diseases are often associated with abnormal immune responses. Beyond autoimmune diseases, HLA‐A also plays a significant role in infectious diseases. For example, the *HLA‐A02* allele is believed to be associated with the prognosis of HIV‐infected individuals, with its presence potentially enhancing the immune response against HIV‐infected cells and improving the clearance ability of cytotoxic T cells [45]. In addition, *HLA‐A* plays a crucial role in tumor immune surveillance, as tumor cells can downregulate *HLA‐A* expression to evade recognition by the immune system, presenting new targets for cancer immunotherapy.

The association between the *HLA‐A* gene and CD has been confirmed in multiple studies. Studies have shown that the *HLA‐A02* and *HLA‐A30* alleles are more prevalent in patients with CD, suggesting that these genes may play a significant role in disease susceptibility [46]. The polymorphisms of *HLA‐A* may influence the functionality of the immune system, altering the body's response to gut microbiota and antigens, thereby impacting the pathogenesis of CD. In the pathophysiological mechanisms of CD, *HLA‐A* molecules present antigens in the gut (such as bacterial and food antigens) to *CD8* ^+^ T cells, activating these cells and triggering an immune response [47]. If the expression or function of *HLA‐A* is altered, it may lead to misrecognition by the intestinal immune system of the normal gut microbiota or food antigens, resulting in a chronic inflammatory response. This abnormal immune response is considered one of the core pathological features of CD.

Furthermore, changes in the expression of the *HLA‐A* gene may also impact the function of intestinal epithelial cells. In patients with CD, the intestinal epithelial barrier is often compromised, and abnormal expression of *HLA‐A* molecules may further weaken the integrity of this barrier, resulting in increased intestinal permeability, allowing antigens in the gut to more easily enter the immune system, thereby exacerbating the inflammatory response [48]. It is noteworthy that the function of the *HLA‐A* gene is not limited to regulating immune responses; it is also closely related to the balance of the gut microbiota [49].

Beyond IBD, *HLA‐A* and closely related *HLA* Class I alleles have long been recognized as key determinants of adaptive immune responses in several clinically important settings. Variability in *HLA* Class I has been associated with inter‐individual differences in vaccine immunogenicity and the persistence of protective antibody responses to viral vaccines such as hepatitis B virus and *SARS‐CoV‐2*, underscoring its role in shaping antiviral immunity [50]. In oncology, HLA Class I genotypes and diversity have been linked not only to susceptibility to certain cancers but also to the efficacy and immune‐related adverse events of immune‐checkpoint inhibitor therapies, suggesting that *HLA‐A*‐driven antigen presentation can influence both antitumor immunity and autoimmunity [51, 52, 53]. Moreover, *HLA‐A* matching between donor and recipient remains a cornerstone of solid organ transplantation, where mismatches or donor‐specific anti‐*HLA‐A* antibodies are associated with higher risks of acute and chronic allograft rejection [54].

Although there have been some MR analyses regarding CD, including studies on oxidative stress gene expression, DNAm, gut microbiota, and mitochondrial DNA copy number, our study is the first to use pQTL and eQTL in MR analysis with CD. This not only provides a more solid foundation for exploring the causal relationship between the *HLA‐A* gene and CD but also effectively avoids potential confounding factors. The CD data used in this study comes from the latest and largest GWAS; the scale and diversity of this dataset significantly enhance the statistical power of the results, improving the understanding of genetic susceptibility to CD. In addition, stringent selection criteria were implemented in the process of choosing IVs. The study also implemented colocalization and SMR analyses, further enhancing the robustness of the results. Colocalization analysis can reveal whether the causal relationship between the *HLA‐A* gene and CD is influenced by other genes, while SMR analysis strengthens the reliability of causal inference by summarizing data. The combination of these two analytical methods provides strong support for the conclusion that *HLA‐A* is a genetically validated immune susceptibility marker in CD.

This study has several limitations. First, the MR analysis of drug targets assumes an ideal condition of lifelong low‐dose drug exposure. However, the reality is more complex, as drug efficacy may be influenced by various external factors. Although this study provides important insights for the development of new drugs for CD, the ultimate efficacy of these drugs still needs to be validated through clinical trials. Second, MR analysis can only independently assess the impact of single druggable gene expression on the outcomes of CD. Many drugs exert their therapeutic effects through the synergistic action of multiple targets; therefore, analyzing the *HLA‐A* gene in isolation may not adequately reflect its true role within complex biological systems. This limitation may affect our comprehensive understanding of the role of the *HLA‐A* gene in CD. Furthermore, the data used in this study is primarily based on eQTL data from blood, lacking specific eQTL data from intestinal tissues. In the context of CD, biomarkers from the gut are released into the bloodstream; while blood can partially reflect the dynamic interactions of systemic processes, it also poses risks of dilution and potential masking of tissue‐specific signals. Finally, the participants in the GWAS were exclusively of European descent, which may limit the applicability of the results to other ethnic groups. Recent trans‐ancestry analyses have shown that the genetic architecture of CD exhibits notable ancestry‐dependent differences in risk allele frequencies and effect sizes (e.g., *NOD2* and *TNFSF15*) and that polygenic scores trained in European populations transfer suboptimally to east Asian cohorts.

## 5 Conclusion

This study has opened a new avenue for identifying immune susceptibility markers through MR analysis based on druggable genes. Our findings suggest that the *HLA‐A* gene represents a genetically validated immune susceptibility marker in CD, providing a refined understanding of the immune genetic architecture underlying this disease. Nevertheless, further experimental evidence is needed in the future to validate the specific role of *HLA‐A* in CD and its clinical application potential, with the aim of providing more effective treatment strategies for patients with CD.

## Funding

The author has nothing to report.

## Conflicts of Interest

The author declares no conflicts of interest.

## Supporting information


**Table S1:** Instrumental variables for pQTL druggable genes.
**Table S2:** Results of MR analysis and sensitivity analysis of pQTL and CD.
**Table S3:** The result of colocalization analysis.
**Table S4:** Instrumental variables for eQTL druggable genes.
**Table S5:** Results of MR analysis and sensitivity analysis of eQTL and CD.
**Table S6:** Results of SMR analysis.

## Data Availability

The data that support the findings of this study are available from the corresponding author upon reasonable request.
